# Legros and Onimus upon the Influence of Electrical Currents upon the Nervous System

**Published:** 1870-06

**Authors:** 


### Original Translations.

ARTICLE I.—*Legros and Onimus upon the Influence of Electric Currents upon the Nervous System.* From the Journal de l'Anatomie et de la Physiologie. (Robins.)

[Continued from May No., 1870.]

§ 4.—*The Influence of Continuous Currents Acting at once upon the Nerves and upon the Muscles.*

Thus far we have studied the influence of electric currents in cases in which the reophores are placed directly in contact with the denuded nerves. These conditions are very simple for experimental purposes, but never exist for medical applications, as the electric current is never applied directly upon the nerves, these being always covered with a layer, more or less thick, of the different tissues.

In the application of electricity to man, two cases may be distinguished, the one in which the reophores are placed along the track of a nerve, and in regions in which it is very superficial, and the other in which one of the reophores is placed along the track of the nerve and the other upon the muscle to which this nerve is distributed. In the case in which the reophores are placed along the course of the nerve, the condition of direct action upon the peripheral nerves is established; but as the nerve is not in immediate contact with the electrodes, the products of the electrolysis do not accumulate there, and it is thus, to a great extent, protected from the chemical actions which the electric currents might determine upon the nervous filaments.

It remains for us to study the influence of electricity in that case in which it acts at once upon the nerve and the muscle.

If an electric current is passed from one leg of a frog to the other — not skinned, but from which the head has been separated from the trunk, in order to prevent all voluntary action — it will be perceived that there can be no longer obtained, but with great difficulty, the alternating contractions referred to already. Whatever may be the direction of the current, it is always at the moment of closing the current that the most energetic contractions are obtained. The strongest and most complete contractions no longer occur, in this case, in the limb traversed by the direct current, but, on the contrary, in that traversed by the ascending current. It is at the closing, and not at the opening, that these contractions occur, and they continue still under the form of partial or fibrillary contractions, during some time after the closing of the current. When the animal commences to be fatigued, the contractions reappear at the opening of the current.

If the spinal cord be diminished (?) or destroyed, or if the animal be poisoned by narcotics, the contractions which were originally very strong in the limb traversed by the ascending current, diminish very perceptibly in energy and in extent; they are then more decided in the opposite limb.

In applying electricity through the skin of robust animals, generally, energetic contractions are obtained only at the moment of closing the current. The same is true of man.

In man, in pathological cases, in which sensibility is found to be diminished, the most energetic contractions are determined equally by employing a descending current, whilst in the normal state, and especially in very excitable persons, it is by means of the ascending current that the most vigorous contractions are obtained.

In cases of anæsthesia, the ascending current occasions very feeble contractions. These facts, which we have several times verified, and which will receive still farther confirmation from those which we are about to study, when we come to examine the influence of electric currents applied directly to the cord, prove, in a very clear manner, that the ascending current determines reflex contractions in the muscles of the limb which it traverses.

As we have already said, it is the ascending current which acts most energetically upon sensitive nerves, and it is known, on the other hand, that excitation of the sensitive fibres determines reflex contractions.

Every reflex action is effected by the intermediation of the cord; but there is another circumstance, which was forgotten among the facts just stated, which permits a portion, at least, of these contractions to be considered as being not *reflex* but *induced.* It is known, indeed, that the electrical excitation of a nerve determines, in another nerve in contact with it, a change of condition, which manifests itself by the contraction of the muscle to which the nerve remains attached. This fact, discovered by M. du Bois Raymond, has received the name of induced contraction; it may be appropriately enunciated thus: *the state of activity in a nerve may induce the same state in a neighboring nerve, although they may not be connected by a nervous centre.* This proposition, thus enunciated, appears very bold, and moreover, it is the real expression of a phenomenon discovered by experiment.

Indeed, in applying an electric current to the nerve of a muscle, the only portion of the nerve through which the electric current circulates is that embraced between its points of contact, and the contraction of the muscle occurs not because the electric current reaches the muscle, but because the nerve is excited at one of these points. All that portion of the nerve between the muscle and the nearest point of contact of the current is therefore not traversed by the current: in this portion, therefore, the nerve is at rest, and it is the change of condition experienced by this portion which determines the excitation of the motor nerve of the muscle. Indeed, as may be seen later, it is possible to excite in this case the electrotonic state of that portion of the electrized nerve lying between the muscle and the nearest point of contact of the current, but that fact does not contradict what is here asserted, that is to say, the induced contraction of muscles by the excitation of sensitive nerves alone, the impression not having traversed the spinal centre. We cannot examine here the different physiological or pathological conditions in which this proposition could be considered, but, regarding exclusively the subject now under consideration, we believe that this experimental fact ought to be taken into consideration in the conditions of electrization of mixed nerves. Moreover, when the cord is destroyed, contractions in the limb traversed by the ascending current do not become weakened immediately, although it is after the expiration of only a very short interval that this phenomenon is manifested, and it is permitted to assume that, during the first moments in which contractions, still energetic, are maintained, they are due to the direct influence of the sensitive upon motor nerves. The last paragraph may be epitomized by the following conclusions:

*Whenever nerves and muscles are acted on at the same time, the strongest contractions always take place at the moment of closing, whatever may be the direction of the currents.*

*In the limb traversed by an ascending current, the contractions are most energetic when sensibility is preserved; when this is abolished or diminished, the contractions, under the influence of an ascending current, are very feeble. They are, then, much stronger when a descending current is employed.*

*The contractions, under the influence of an ascending current, are due to reflex action, and probably also to the phenomenon observed by M. du Bois Raymond, and termed induced contraction.*

§ 5.—*The Electrotonic State, or Electrotone.*

The effect determined in a nerve by the passage of a continuous current remains to be investigated. If a constant galvanic current is made to pass through some fraction of a nerve, this undergoes, throughout its entire length, a change of state, which is manifested by an increase or a diminution in its own electric current. If the current of the pile has a direction similar to that of the nervous current, the latter is increased in intensity; it is, on the contrary, diminished if the direction of the two currents is inverse. M. du Bois Raymond has applied to this phenomenon the name *electrotonic state* of the nerve. He distinguishes two different phases of this condition, that during which the nervous current undergoes an increase of intensity, which he calls *positive*, and that during which it undergoes a diminution, which he terms *negative*. Then, at the moment when a voltaic current is made to pass through a portion of a nerve, a current in the same direction is produced throughout the whole length of the nerve, both above and below the electrized point. This current is rendered sensible by a galvanometer placed at any point of the nerve. This electric current, which is formed thus, outside of the points directly electrized, had been obtained, until very recently, only in nervous fibre. It might thence be logically concluded that this was a special phenomenon of nerves, and that it was in intimate relation with their functioning. M. du Bois Raymond admits that the electrotonic state is due to a molecular polarization of the nerve analogous to that which is determined in all conducting bodies by the effect of the passage of an electric current. This polarization consists in this, that the nervous molecules, endowed with two electric poles, turn all their positive poles from the side toward which the current is directed, and their negative from the side at which it enters. Under the influence of an exterior current, the nervous molecules are arranged in regular succession, in the manner designated under the name "polarization," and assume this arrangement, even in the portions of the nerve not traversed directly by the current. The result of which is the circulation in the nerve of a new current, which must augment or diminish, according to its direction, the nervous current itself.

Before referring to the experiments of Matteucci, which seem to reduce these different states to simple chemical action, we should again relate the facts discovered by M. Pflüger.

According to this physiologist, a voltaic current, passing through a determinate length of nerve, decomposes the nerve into two different portions, regarded from a physiological stand-point. The portion of the nerve in contact with the positive pole (anode), loses its excitability, and this state has received the name of *anelectrotonic;* near the negative pole (cathode), the excitability is, on the contrary, augmented, and this state is designated by the name *catelectrotonic.* These changes of condition are found, not only at the points of contact of the poles, but likewise in their neighborhood; moreover, they are observed especially with moderate currents. They augment with the intensity of the current, up to a certain maximum, next diminish, disappear, and pass into opposite effects.

Such is the fundamental experiment of M. Pflüger. Whilst a current traverses a portion of the nerve, he moistens the nerve with salt water, which, in its normal state, constantly provokes muscular contractions; on the side of the positive pole, the salt water determines no contraction, whilst it determines them when it is placed in contact with the portions of the nerve approximating to the negative pole. With an ascending current, that is to say, when the negative is placed farther from the muscles than the positive pole, the same effect is obtained, which demonstrates that the excitability determined at the negative pole is sufficiently strong to reach the muscles by passing through a portion of the nerve traversed by a current, and rendered inexcitable by it in one point.

M. Bezold has repeated these experiments, and has epitomized the results which he obtained, in the following propositions:

In nerves, as in muscles, the current passing into these organs in a constant manner, produces, during all the time of its passage, a molecular polarization. This molecular state determines direct excitation only near the negative pole, and the nerve, like the entire muscle, is only excited indirectly by the irritation produced at the negative pole.

At the moment of the opening of the current the excitation takes place directly, only near the positive pole, and the parts which approximate to the negative pole are placed in a state of irritation by the influence of the excitations originating at the positive pole. In other words, at the closing of the current the excitation is due to the constant departure of the current, and at the opening, a state of irritation is induced, during some time, by the perturbations of molecular equilibrium.

M. Bezold adds, and we insist upon this fact, that the excitant effect of a constant current is due, probably, to chemical actions produced by the passage of the current, and that electric excitation is only a determinate form of chemical irritation.

The question of molecular polarization being hypothetical, we will not permit ourselves to be drawn into discussions of this sort, but, from the conclusions of Bezold, it results that, during the passage of the current, the centre of irritation is located near the negative pole, whilst at the opening, the regions in contact with the positive pole become the cause (source?) of excitations, and finally that these phenomena are probably due to chemical action. These conclusions approximate nearly to those of Matteucci, who made, during the later years of his life, very important investigations in relation to the electrotonic state.

In order to explain the theory of Matteucci, we are obliged to reconsider the question from its origin; and, moreover, this discussion is too important for us to be able to summarize it in a few lines.

It is known that when certain inorganic bodies, such as platinum, are electrized, there are formed at the same time *secondary currents*, directed inversely to the principal current. These currents are due to the chemical actions which take place, on the one hand, between the products of the electrolytic decomposition gathered upon the metallic electrodes, and, on the other hand, with the liquid which is in contact with the electrodes. The products of decomposition, as may be ascertained by the aid of litmus paper, are, at the negative pole, alkaline, and at the positive, acid. When the liquid which bathes the electrodes is pure water, the products of decomposition are, oxygen at the positive pole, hydrogen at the negative. Now, in this case, the secondary currents may still be formed, as is proved by the following experiment, due to Matteucci: A wire of platinum, well depolarized, is introduced by one of its extremities into a test tube filled with hydrogen gas. After having left this tube, during some hours, in contact with the gas, if it be next applied to the galvanometer, a deviation of the needle will be obtained.

If, in place of a platinum wire, there be substituted a cord, moistened with salt water, a cylinder of clay, saturated with the same, a fragment of vegetable or animal matter, as slices of potatoes, stalks of celery, muscles, nerves, electric phenomena of the same character will be likewise obtained. Of all these substances, nerves possess this property to a very high degree.

"I select," says Matteucci,* "in a chicken or a rabbit, which has just been killed, the sciatic nerve, at least 60 to 80 millimetres. I assume it to have been already tested experimentally that this nerve, placed upon the cushions of the galvanometer, exhibits no indications of an electric current. I then take this nerve and place it upon platinum electrodes in such a position that the extent traversed by the current between the electrodes may be 25 or 30 millimetres, and that two ends of the nerve, each about 15 to 20 millimetres in length, remain hanging outside of the electrodes. Then I transmit the current from a little pile of not more than eight or ten elements, during a period not exceeding a fraction of a second, and which, ordinarily, I continue from thirty to sixty seconds. After this passage, I quickly transfer the nerve to the cushions of the galvanometer: immediately I obtain a strong deviation, which depends upon the duration of the passage of the current, and upon the power of the pile, and which, in every portion of the nerve situated between the poles, indicates a current contrary to the current of the pile. I find, likewise, indications of a current, upon touching outside of the poles — that is to say, between the points of the nerve which were in contact with the negative pole, and with the positive, and which were near, and at points most distant, or neutral — which had not been in the track of the current of the pile. In these portions situated outside of the electrodes, the secondary currents have the same direction as the current of the pile, and experiment has demonstrated that the strongest secondary current is always that at the end of the nerve which is beyond the negative pole. It has been recognized, likewise, that by prolonging considerably the passage of the current, or by using a weaker current, the secondary currents obtained in the exterior parts all take, finally, the same direction as the intermediate current; that is to say, by being at all points, both between the electrodes and outside of them, contrary to the current of the pile. If we recall, here, the known principles of electro-chemistry — that is to say, that there is always an electric current produced at the point of contact between a base and an acid, between the water and the acid, between the base and the water, a current which goes directly from the base to the acid, from the water to the acid, from the base to the water — we will under-

* *L'electro-physiologie.* (*Revue des cours Scientifiques*, 8 août, 1868.)

stand, without difficulty, how these secondary effects are developed in nerves after they have been traversed by electricity, from the moment when we know that at the contact of the electrodes the nerves gather the products of the electrolysis."

In order that secondary currents may be obtained, it is not necessary that the nerve be fresh, it suffices that it be moist, and that its structure be not altered; these phenomena occur still in the nerves of animals dead several hours, and in nerves plunged during several hours in water at 40 degrees of temperature. They disappear after the nerve has been compressed at any point in such a manner as to destroy the homogeneity of the axis cylinder. Matteucci supposes, after experiments made upon bodies such as moist chalk, pieces of potato, which give secondary currents much more energetic when they are traversed in the direction of their axis by a metallic wire, that the axis cylinder plays, in the nerve, the role of a better conducting body, and that it is upon this that the products of electrolysis are deposited.

Lastly, the experiment of Matteucci consists in covering with two layers of hempen or cotton threads a platinum wire of one metre in length and one millimetre in diameter; these threads are moistened with salt water, and arranged consecutively, as in the experiment upon electrotone, that is to say, with one of their extremities placed upon the cushion of the galvanometer, separated from each other by a distance of 20 to 25 millimetres, whilst the other rests upon the electrodes of the pile, or upon the cushions communicating with the electrodes. The current is scarcely formed, when a strong electrotonic current is obtained, even at the distance of 30, 40, 60 centimetres, and more, from the electrodes of the pile.

By applying to the platinum wire, thus covered, litmus papers, the paper will be seen to redden at the points of contact of the positive electrode; outside of it, however, and even at a great distance from these points, the paper becomes blue, and consequently indicates an alkaline reaction. These entirely opposite effects occur near the negative pole.

As to the phenomena observed by Pflüger, consisting of a loss of excitability near the positive pole, and an augmentation of excitability near the negative, Matteucci explains them by this fact, that in contact with the negative pole the nerve is loaded with hydrogen and alkaline matter, and that it is loaded, in contact with the positive pole, with oxygen and acid. Now, as Humboldt first discovered, the excitability of the nerve is augmented when it is kept in contact with a very weak alkaline solution, whilst it is weakened in contact with an acid solution much diluted.

According to Matteucci, the phenomena of electrotone, and those of the catelectrotonic and anelectrotonic states, are then produced only by the chemical actions which are developed by the passage of the current. It is important to compare this opinion of Matteucci with the conclusions of Bezöld, which, although less definite and clear, seem to give the same results.

We accept very willingly the greater portion of the theories of Matteucci; however, whilst recognizing that they are certainly true, we believe that they are so only in part, and that they are insufficient to explain all the phenomena.

The most serious objection in this identity between the electrotonic state and the secondary currents is the rapidity of transmission of electrotone, for this transmission is equal, not to that of electricity, but, indeed, to that of nervous influx; and, just as this condition is one of the best arguments to demonstrate that nervous influx cannot be identified with electric phenomena, we ought, in this case, to admit equally that these phenomena cannot be identified with the electrotonic state.

Moreover, if electrotone was a phenomenon purely physical, the force of the current would not act differently according to its intensity, and it has been shown that, far from augmenting with a strong current, the electrotonic state diminishes, and disappears completely.

Lastly, the electrotonic state, that is to say, the loss of excitability near the positive pole, is produced without the direct application of the electrodes to the nerves: indeed, in several cases, a diminution of excitability is observed in the neighborhood of the positive pole, when the latter is separated from the nerves by a thick layer of epidermis and adipose tissue.

The experiments of Matteucci remain no less complete, and possess great importance, although we believe, according to our own experiments, two cases should be distinguished, that in which the nerve possesses all its excitability, and that in which the nerve no longer retains its excitability, and acts as a conducting body, susceptible of undergoing the phenomena of electrolysis.

In the first place, every current, whatever may be its properties, its origin, and its direction, determines an excitation of the nerve to which it is applied. The nerve, fresh and living, assumes an active condition at the least disturbance, the slightest irritation, whether this irritation be mechanical, chemical, or electrical; hence, it is evident that the electric current, which modifies profoundly the molecular condition of bodies which it traverses, will determine nervous activity, and that, too, whether the current be ascending or descending, and at the moment of the closure as well as at the moment of the opening, for in each of these moments the molecular state is modified.

But at the end of some time, when the nerve has been exposed to the air, or has been fatigued by the current, its excitability disappears, and here, then, are interposed different conditions and phenomena, due, not to the direct action of the current upon a portion of the electrized nerve, but to the action of derived currents and currents of polarization.

